# Syphilis Trigram: a domain-specific visualisation to combat syphilis epidemic and improve the quality of maternal and child health in Brazil

**DOI:** 10.1186/s12884-022-04651-w

**Published:** 2022-05-01

**Authors:** Cleber Matos de Morais, Igor Vitor Teixeira, Sara Sadok, Patricia Takako Endo, Judith Kelner

**Affiliations:** 1 Centro de Informática, Universidade Federal de Pernambuco, Recife, Brazil; 2 Departamento de Mídias Digitais, Universidade Federal da Paraíba, João Pessoa, Brazil; 3 Programa de Pós-Graduação em Engenharia da Computação, Universidade de Pernambuco, Recife, Brazil; 4 Programa de Pós-Graduação em Engenharia da Computação, Universitat Autònoma de Barcelona, Barcelona, Spain

**Keywords:** Syphilis, Congenital syphilis, Data visualization, Poverty

## Abstract

**Background:**

The Brazilian healthcare system is a large and complex system, specially considering its mixed public and private funding. The incidence of syphilis has increased in the last four years, in spite of the presence of an effective and available treatment. Furthermore, syphilis takes part in a group of disorders of compulsory notification to the public health surveillance. The epidemiological implications are especially important during pregnancy since it can lead to complications, related to prematurity stillbirth and miscarriage, in addition to congenital syphilis, characterized by multisystem involved in the newborn.

**Methods:**

The Action Research methodology was applied to address the complexity of the syphilis surveillance scenario in Pernambuco, Brazil. Iterative learning cycles were used, resulting in six cycles, followed by a formal validation of an operational version of the syphilis Trigram visualisation at the end of the process. The original data source was analyzed and prepared to be used without any new data or change in the ordinary procedure of the current system.

**Results:**

The main result of this work is the production of a Syphilis Trigram: a domain-specific infographic for presenting gestational data and birth data. The second contribution of this work is the Average Trigram, an organized pie chart which synthesizes the Syphilis Trigram relationship in an aggregated way. The visualization of both graphics is presented in an Infographic User Interface, a tool that gathers an infographic broad visualization aspect to data visualization. These interfaces also gather selections and filters tools to assist and refine the presented information. The user can experience a specific case-by-case view, in addition to an aggregated perspective according to the cities monitored by the system.

**Conclusions:**

The proposed domain-specific visualization amplifies the understanding of each syphilis case and the overall characteristics of cases of a chosen city. This new information produced by the Trigram can help clarify the reinfection/relapse cases, optimize resource allocation and enhance the syphilis healthcare policies without the need of new data. Thus, this enables the health surveillance professionals to see the broad tendency, understand the key patterns through visualization, and take action in a feasible time.

## Introduction

Sexually Transmitted Infections (STI) are considered a public health problem and are among the most common transmissible diseases [1], negatively affecting people’s quality of life and health. Among them, syphilis is a systemic infection exclusive to humans caused by the spirochete *Treponema pallidum*, transmitted in three ways: sexual, congenital, or via blood transfusion. Sexual transmission is the predominant route of transmission [2], followed by congenital. The latter is the result of the transmission of *Treponema pallidum* present in the bloodstream of the pregnant woman to the conceptus via the placenta, also known as transplacental infection or, occasionally, through direct contact with the infectious lesion at the time of delivery.

The adverse outcomes of untreated gestational syphilis are early pregnancy loss (40%), fetal death (11%), and preterm or low weight birth (12% to 13%). Furthermore, at least 20% of newborns have signs suggestive of early congenital syphilis [3]. In newborns, congenital syphilis can have early or late manifestations. The first is defined by the presence of symptoms occurring before two years of age, as opposed to the second, characterized by the onset of symptoms after the referred time frame. The main consequences of early congenital syphilis are preterm and impaired fetal growth, in addition to the most common symptoms - hepatomegaly, jaundice, nasal discharge, rash, generalized lymphadenopathy and skeletal abnormalities. In addition, a miscellaneous of skin lesions, diaphyseal periostitis, radiographic abnormalities, limb pseudoparalysis, also known as pseudoparalysis of Parrot, respiratory distress, among other consequences can also be present. In late congenital syphilis, on the other hand, due to the persist inflammation generated by the treponemas, the constellation of symptoms include facial features, such as saddle nose, short maxilla and protuberant mandible. Moreover, neurological manifestations, such as intellectual disability or cranial nerve palsies might occur. Skeletal system involved is characterized by saber blade tibia and anterior bowing of the shins. Concerning the oropharynx, incisor teeth (Hutchinson’s teeth) and blackberry molars can occur. Also, interstitial keratitis and sensory hearing loss - the latter, normally diagnosed at 6-8 years of life. [2]. The efforts towards preventing the vertical transmission is justified when one considers all the severe consequences of the affected newborn [4]. Considering that the condition has an easy and accessible treatment, it is objectively eradicable.

According to Pinto et al. [5], there was a gradual increase regarding the number of syphilis tests from 2015, in which the amount of tests was 518,859. In 2016, this number increased to 911,420 tests, and from 2017, there was a more apparent rise, having 1,448,364 tests in 2017, 2,068,184 tests in 2018 and 2,533,571 in 2019. This scenario allowed a better understanding of situation of gestational and congenital syphilis in Brazil. According to the Syphilis Epidemiological Bulletin 2020 of the Ministry of Health of Brazil [1], Brazil has been facing a syphilis epidemic since 2016, despite being a treatable disease with an affordable and low-cost medication, such as penicillin. In 2019, the syphilis detection rate in Brazil was 72.8 cases per 100,000 population; the detection rate of syphilis in pregnant women was 20.8/1,000 live births; the incidence rate of congenital syphilis was 8.2/1,000 live births and the mortality rate from congenital syphilis was 5.9/100,000 live births [1].

**Fig. 1:**
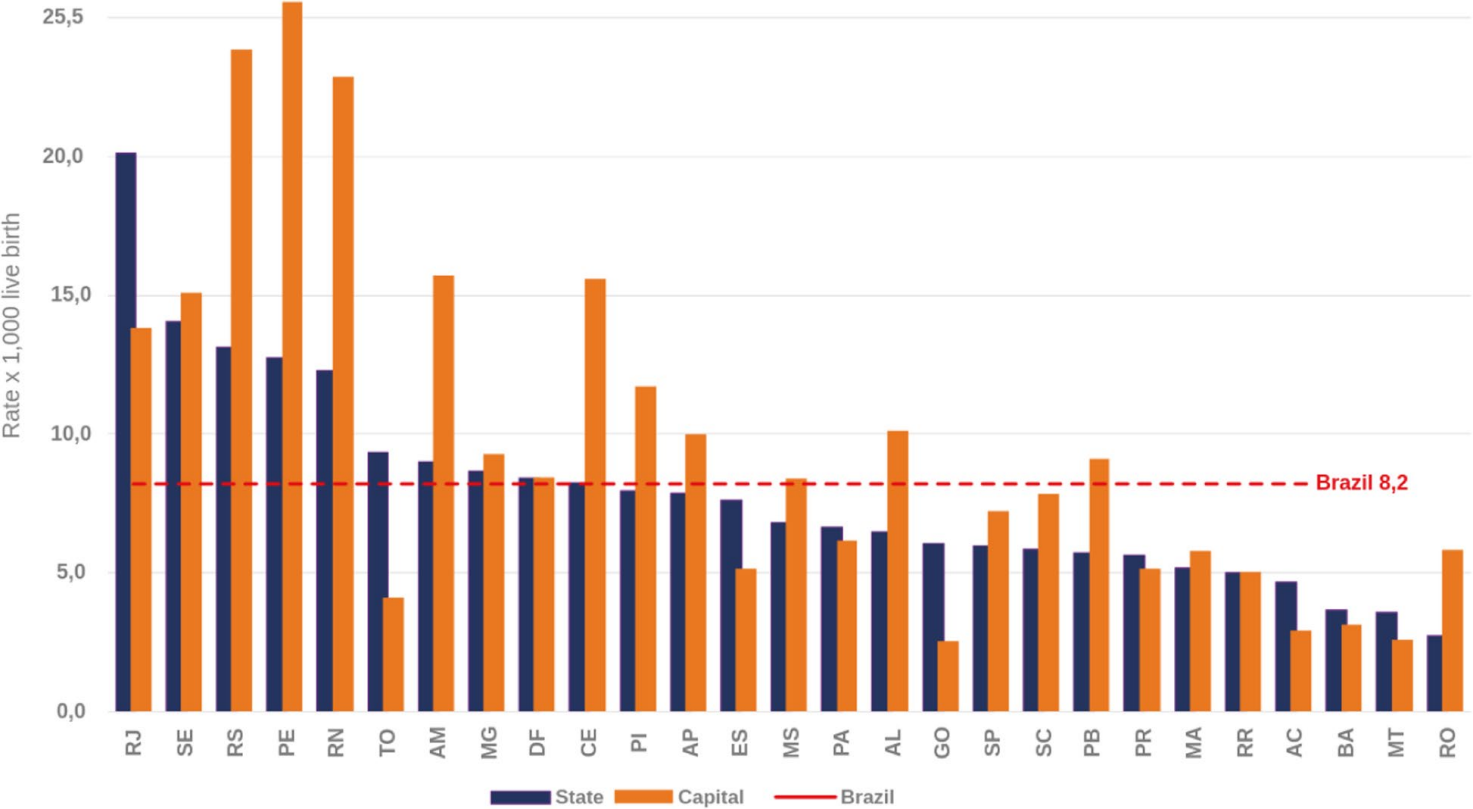
Incidence rates of congenital syphilis (per 1,000 live births). Brazil, 2019. Source: Sistema de Informação de Agravo de Notificação (SINAN), updated on 06/30/2020

**Fig. 2:**
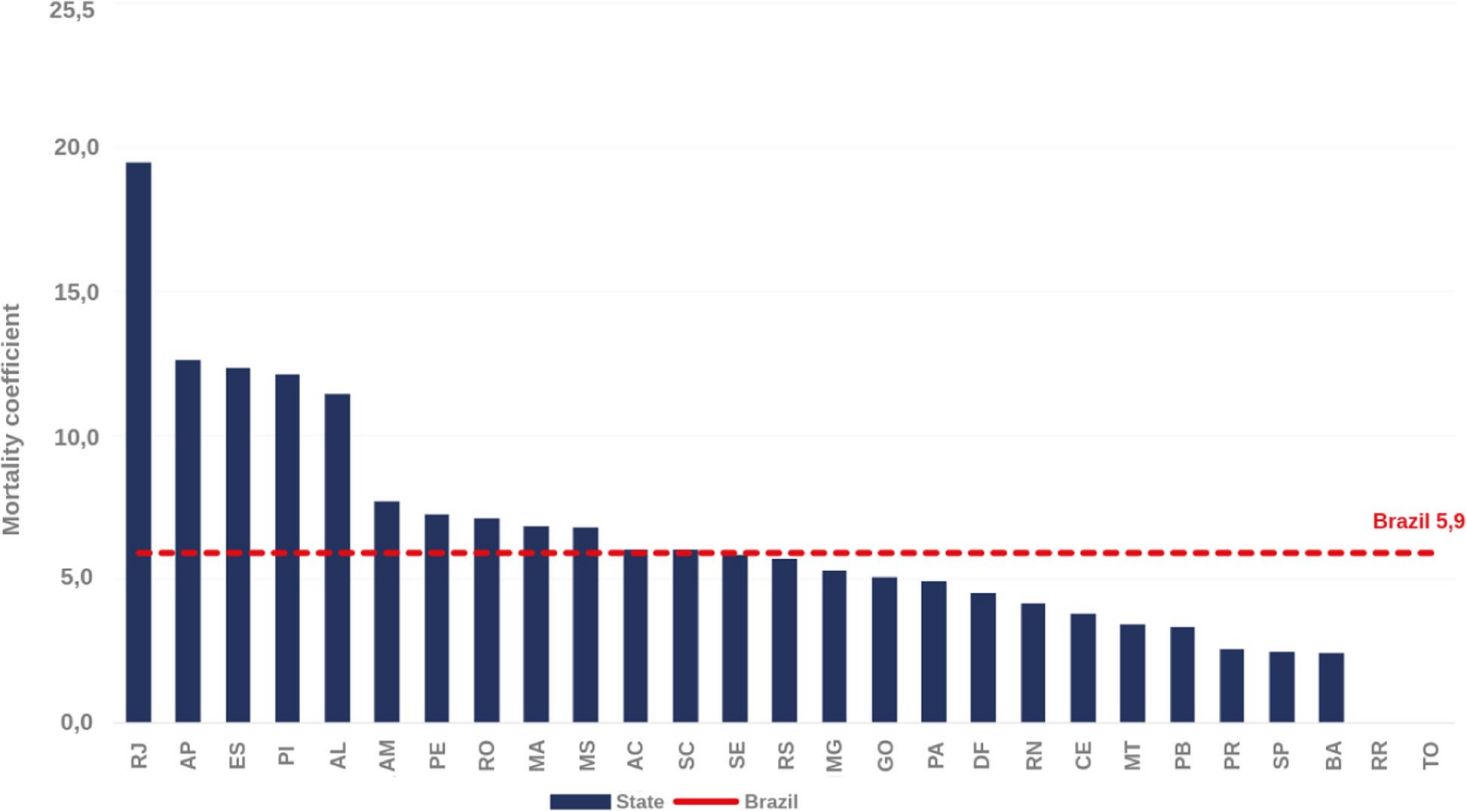
Infant mortality coefficient due to congenital syphilis (per 1,000 live births). Brazil, 2019. Source: *Sistema de Informação sobre Mortalidade* (SIM), updated on 06/30/2020

In the last ten years, there has been a progressive increase in the incidence rate of congenital syphilis, and 2019 was the first year along this temporal series to show a reversal [6] due to the enforcement of public health intervention policies and projects like “Syphilis No!”, responsible for the production of campaigns to prevent syphilis and to eliminate the transmission of congenital syphilis. The city of Recife, in the impoverished Northeast region of Brazil, was the one with the highest incidence rate in 2019 (with 25.6 cases/1,000 live births), a rate three times higher than in Brazil (Fig. 1). Considering the mortality from congenital syphilis in children under one year per 100,000 live births, the state of Pernambuco occupies the 7th place, with a rate of 7.2 (Fig. 2).

Despite the numbers presented and unlike many neonatal infections, congenital syphilis is a preventable condition, as long as there is identification of the infection during pregnancy (through effective prenatal care) and appropriate treatment is carried out. According to Macedo et al. [7], “*with relatively simple and oriented interventions for the case of mothers and newborns, it is possible to obtain a great reduction in congenital syphilis*”.

In this context, it is necessary to create and update public policies in the area of maternal and child health to improve the current situation. There are already several efforts at different levels to reduce maternal and child deaths with the public administrators. Among these efforts, there are specific guidelines for assistance to pregnant women infected with syphilis (and other STI) in Primary Health Care (PHC). For example, there is the Mãe Coruja Pernambucana Program (PMCP) [8], a Brazilian prestigious social program in the maternal and child area, implemented in October 2007, recognized and awarded by the United Nations (UN) and by the Organization of American States (OAS), as a Public Policy Management model. The PMCP is a priority program of the government of Pernambuco, which aims to ensure comprehensive care to pregnant women using the Sistema Único de Saúde (SUS), the Brazilian public health system, and their children up to 5 years old, creating a solidarity network to reduce maternal and child mortality, in addition to contributing to the improvement of social indicators. Currently, the PMCP is present in 105 most vulnerable municipalities in the state of Pernambuco^1^.

However, the scarcity of communication and information channels, and the stigma regarding STI in society, especially in the context of vulnerable populations, weakens awareness and access to preventive measures and, consequently, to treatment for these diseases. According to Macedo et al. [7], “*among the sociodemographic factors, low education, low income and material status (stable or non-stable union) are identified as risk situations and an expression which syphilis is related to poverty, although it is not limited to it*”. Additionally, although the rapid test for syphilis is available free of charge to the entire Brazilian population [9], its scarcity in PHC is common and can contribute to masking the real number of positive diagnoses, leading, consequently, to a decrease in the number of patients who should undergo treatment and follow-up. [10].

Regarding the state-of-the art, Rosenbaum et al. [11] and Doherty et al. [12] used graphs to represent the social networks of syphilis transmission. Due to the pattern of the transmission, this analysis of social networks can contribute to the understanding of the propagation process in a given geolocation. Fede et al. [13] applied a geospatial representation called Ring Maps that shows, by region, an indication of the presence of cases of syphilis, chlamydia, Human Immunodeficiency Virus (HIV) and gonorrhoea. The visual synthesis adds an innovative perspective, including the possibility of representing demographic data (such as ethnicity and unemployment level) to associate with the quantitative result of the region. Sullivan et al. [14] proposed a new type of scatter plot that relates the rates of primary and secondary syphilis by ethnicity in men who have sex with men, sorting by location and sub-location. In terms of variable correlation, it is an effective visualization of the relationship between two rates, proportionality of an ethnicity, locality and sub-locality. However, no previous work in the literature presented a visualization with a narrative of the individual data of the pregnancies that was able to produce quality information to understand and/or raise awareness for cases of reinfection, recurrence or lack of testing.

According to Santos et al. [10], knowing the trends of syphilis’ cases and identifying the main factors related to PHC and the sociodemographic structure of a locality can guide new strategies for health promotion and disease prevention, as well as direct resources that significantly influence the reduction of a possible epidemic. In this way, the main goal of this work is to present the Syphilis Trigram, a visualization system that represents the narrative of individual pregnant women with syphilis. The solution offers quality information for analysis, comparisons with a short learning curve, giving inputs to improve the care and follow-up of syphilis in pregnancies and to understand cases of reinfection, recurrence or lack of exams. Moreover, the Syphilis Trigram is applicable in data provided by the PMCP without the need to add or modify the primary data.

## Methods

### Context and data

Initially, the data set used in this work was composed of information of four cities of Pernambuco (Aliança, Araçoiaba, Bonito, and Petrolina) from 2016 to 2017, having 1,552 records of pregnancies and 10,185 records of prenatal care. Those cities were selected because they are in different macroregions of the Pernambuco State and an elevated case incidence per 100,000. In order to study the scalability of the solution, another data set covering all cities of Pernambuco served by the PMCP was made available. This data set had information from 2016 and 2017 of 43,938 pregnancies with 217,444 prenatal care, 39,309 births and 38,559 children.

### Data visualization method

Pregnancy data are a time series junctions of social-demographic data with health data from antenatal care. Each antenatal care encounter produces new time-aware data in the context of pregnancy. Even at birth, the newborn data is bound to the mother and all pregnancy data during time. Therefore, considering the multivariable nature of pregnancy data and the need raised by the Customers, Actors, Transformation process,Weltanschauung, Ownership, and Environmental constrains (CATWOE) analysis (that will be presented in the 1st cycle), an infographic perspective was chosen for the visual representation of this system. Infographics are diagrammatic representations of data [15], more complex than a series of data displayed together or a story presented through images. In essence, an infographic representation has a distribution for each piece of information that has purpose and meaning within the visual space [16]. Furthermore, infographics have an exploratory nature through interaction, which is one of the main goals of data visualization [17]. One of the elements favored by interactive infographics is the dual cognitive experience. The human brain responds well to the simultaneous use of motor (hands) and visual (graphics) elements at the same time in the same context [18, 19]. The consciousness part (vision-to-perception) and the call to action (vision-to-action) are activated simultaneously in infographic interfaces. This dual mental stimulus is especially useful in systems that rely on high specificity and precision from users, such as healthcare systems. The ability to communicate large amounts of data, multiple variables visually and allow exploration is critical to the greater usefulness of a healthcare system, enabling the end-user to notice events and act as desired in a complex data environment [18].

Based on the three pillars – data consistency, relevant statistics, and design – we defined the following principles for the infographic visualization system:

1. The data must be appropriate to the user in terms of relevance, quantity, and temporality;
2. The infographics must allow for accurate comparison in the same viewing context;
3. The design narrative must be consistent and meaningful throughout the infographic;
4. The infographic must allow the user to explore, expand and compare data and information in the same interface; and
5. The infographic visualization system must allow refinements to a level of data that the user wants to see, according to their context of use, allowing new visualizations and analysis.

### Action research methodology

This work considers the question addressed from the integral perspective of the Brazilian SUS. The SUS information scenario is complex due to the diversity of users, with different relationships in their functions and data sources that circulate between the systems. This means that the system cannot be considered using an alternative of reductionism (understand the whole system from the individual qualities of the parts) or holism, which simplifies the parts to create a possible view of the whole [20]. Therefore, this research was conducted using an Action Research methodology approach [21] and among the various methodological views of the Action Research, the chosen one was proposed by Peter Checkland, the Soft System [22]. It is a methodology for the analysis and development of systems in which researchers and stakeholders participate jointly in the process of designing and developing the system in an iterative way. The Soft System appears as an alternative to the systems engineering concept of Hard System. Hard Systems are systems which, given an explicit definition of an objective, the conceived system will be engineered for that particular purpose, under various constraints (budget, legal, environmental, etc.). In many scenarios, this approach can be applied to troubleshooting. However, real-world systems with a multi-layered process, like SUS, are much more complex. Another factor for choosing the Soft System methodology is its integrated relationship with the human actors in the systems. For Checkland [21], there is a special kind of system, human activity systems, which is intrinsically linked to human action and because of this cannot be reduced in its complexity to create a model. Systematic human action, as in a health system, does not behave like a natural system or an autonomous system.

**Fig. 3:**
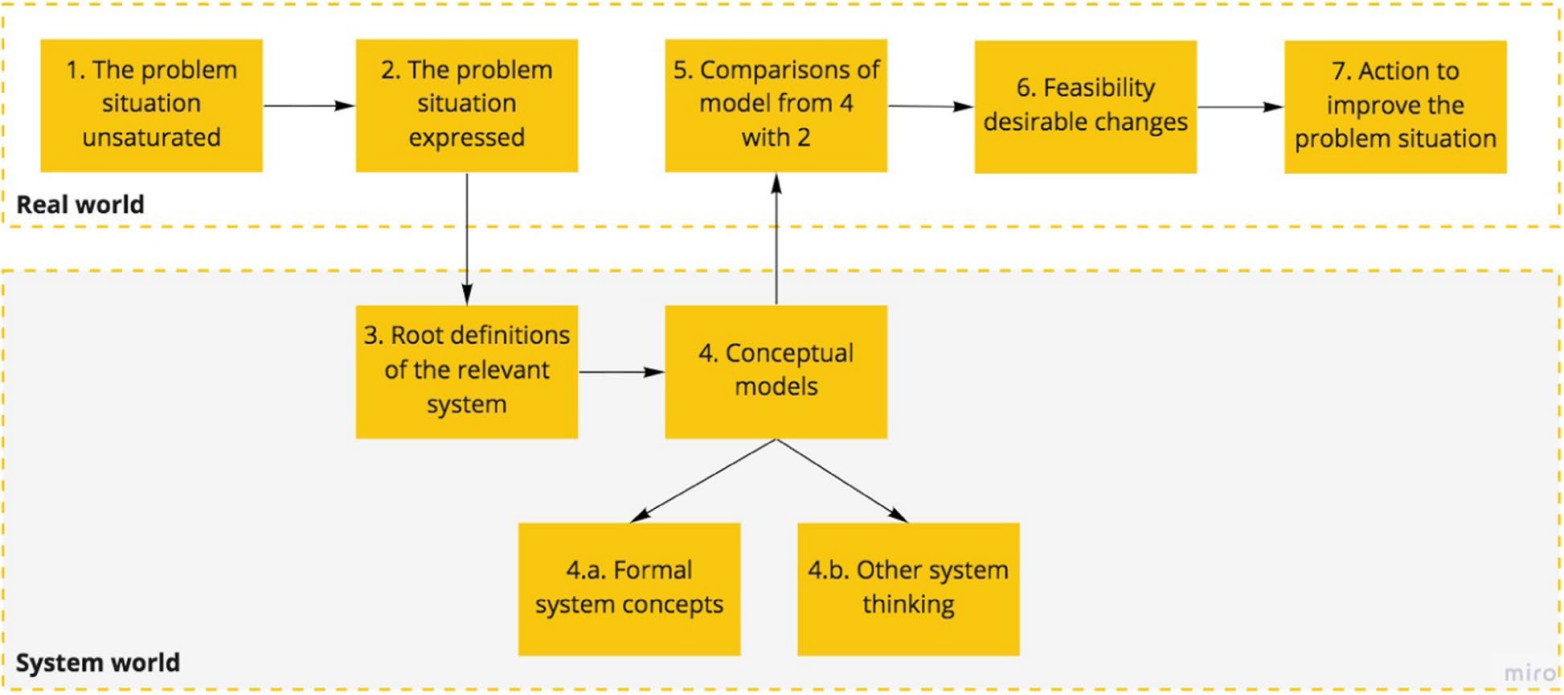
Cycles activities. Adapted from McManus [23]

The analysis for this work is based on Checkland’s iterative learning cycles [21] and the activities of each cycle is presented in Fig. 3. Initially, the production of a clear picture of the business process is necessary. This is a multi-perspective system analysis, with a diagrammatic system representation called “rich picture” as output. Some root definitions are proposed, representing a core need that should meet an actual and specific systematic complexity. After this cycle, the researchers and the stakeholders build together a concept model for the system. This model is validated formally and within the interaction with others related systems. Subsequently, the model is compared with the initial state of the problem and validate if it fits the overall system’s needs. At the end of each cycle, there is small output (new product, tool or concept) that helps towards the solution of issues elucidated in the previous cycle. So this new reality (new rich picture) is the input for the next cycle.

**Fig. 4:**
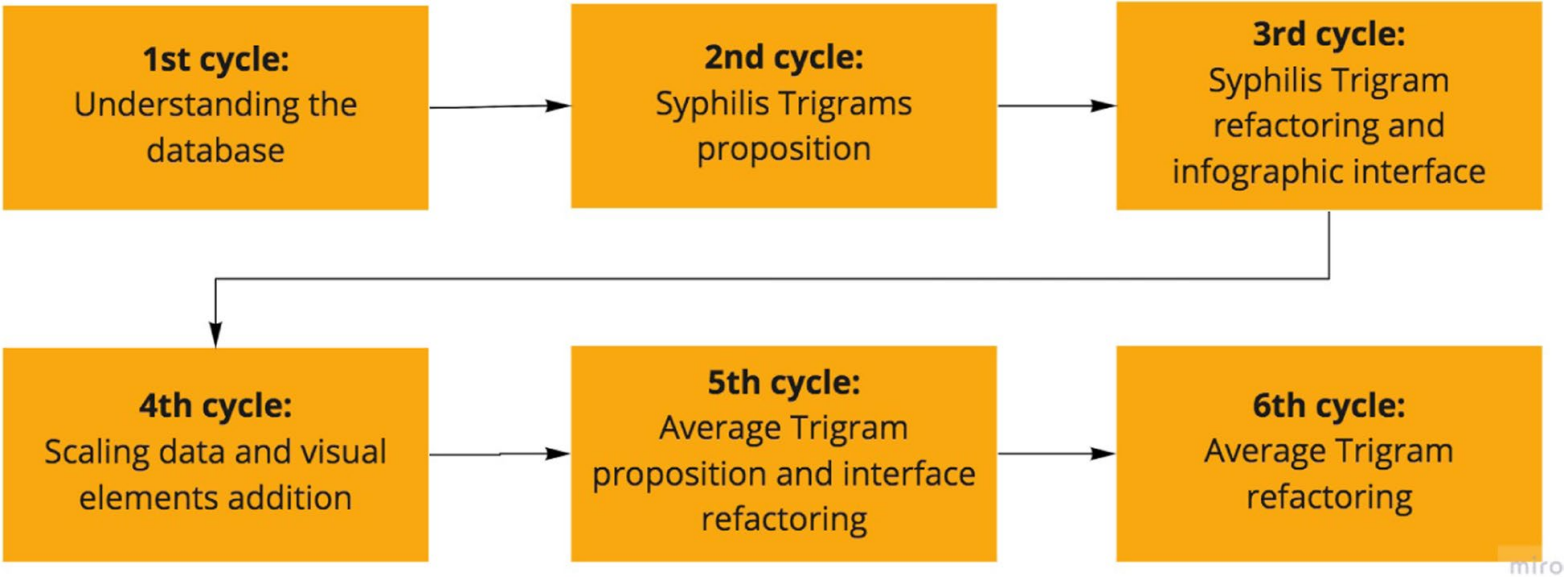
Software System iterative learning cycles

Figure 4 presents the six learning cycles resulting from the interaction between researchers and stakeholders to elucidate and solve the target problem. Each cycle has its specificity and own contributions.

### 1st cycle - data understanding

The first iterative development cycle started with a wish-to-change. This was followed by a stage of description and presentation of the problem and context to understand the entire relationship of use and roles of the stakeholders. This stage was built using two strategies: (i) a CATWOE analysis to create a root definition; (ii) and a mind map of hierarchy relations called a rich picture [24].

The definition of CATWOE elements must be contained within a root definition, so that throughout the development process, it is clear who it is intended for and who evaluates any system resource. The definitions for this first cycle were:

- **(C) Customers:** pregnant women attended by PMCP;
- **(A) Actors:** regional and board managers of PMCP;
- **(T) Transformation:** acquire more information for decision-making in actions to combat syphilis within the scope of PMCP;
- **(W) Weltanschauung:** consider the board managers and data analysts, who have a different view of what the problem is and how to select the data;
- **(O) Owner:** board managers of PMCP; and
- **(E) Environmental constrains:** do not restructure the database or collect new primary data.

Thus, we summarize these elements of CATWOE in the following root definition, defined with the stakeholders: “*Create a visualization system that interprets the current data of pregnant women with syphilis from the PMCP, segmented by social and context variables (gestational risk, age group, race/color, education, income, place of residence, type of population, among others) for production of quality information, without the need to add or modify forms or primary data, for analysts and the board of the PMCP, to help the decision making to improve the care of pregnant women*”.

The main contribution of this investigation would be to identify the variables correlated with the infection of syphilis in pregnant women assisted by the PMCP. In particular, to understand which factors could be more important for the early diagnosis and intervention followed by the proper treatment of syphilis and, therefore, result in faster care for pregnant women with an indicated profile, based on sociodemographic data.

**Fig. 5:**
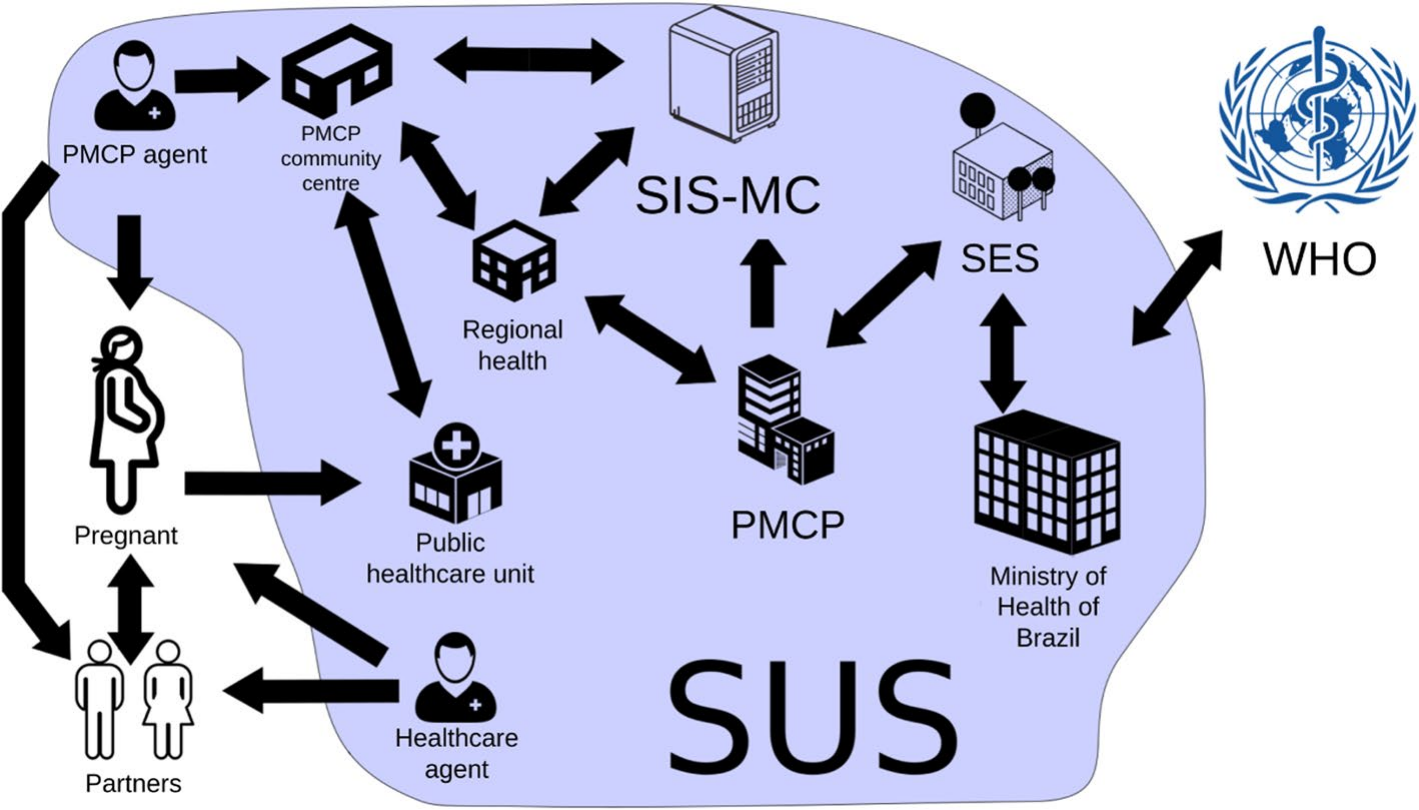
Rich picture of PMCP domain. Source: the author

Rich picture is a strategy of an organic mind map of hierarchies and power relations in the organizational environment. It should be organic by definition, and should not be based on visual models of formal diagrams. In Fig. 5, we have the representation of the PMCP domain, where an important characteristic is that the action is performed by the basic health network. The PMCP team is composed of an articulator and a health care agent, who do not work directly in health care. In addition, the PMCP has hierarchies from the community center to the board of the program.

Accordingly, based on the definition of the root definition, the analysis of data from a sample of the Sistema de Informação do Mãe Coruja (SIS-MC), which is the information and database system from the PMCP, began.

However, even during the conceptual modeling process, it was necessary to create a new root definition. As an alternative, it focused on visualizing data that had a certain level of completeness (greater than 70% of recorded data) and that could be supported by the syphilis test data, both during pregnancy and at delivery. Thus, a new root definition version presented to the stakeholders was: “*Create a visualization system that represents the PMCP narrative about pregnancy with syphilis without the need to add or modify forms or primary data for the analysts and the board of the PMCP, to help the decision making to improve the service to pregnant women*”.

The addition of the narrative concept was crucial in the development of this cycle, as it changed the perspective of punctual information (like in reports with simple queries) to an interrelationship between present and absent data. Focusing on the perception of the pregnancy narrative, we focused on adjusting and recognizing the missing data as information to be considered.

As the main contributions of this first cycle, we can mention:

- Understanding the database and its filling characteristics, data quality, and evolutionary history;
- Understanding that it is not always possible to answer all the questions initially elucidated in the root definition since the quality of data available for analysis must be assessed; and
- Understanding the health domain of the PMCP, especially about specific terms and meanings.

### 2nd cycle - syphilis Trigram proposition

From the result of the data preparation from the first cycle, the process of selecting SIS-MC data for visual representations was started, with the main focus on understanding syphilis cases. Data selection and categorization depends on the presence of syphilis in some of the pregnancy until the outcome. The main expected contribution of this cycle will be an application of domain data for a visualization that represents the dynamics of syphilis.

The elements of the CATWOE analysis remain the same as in the first cycle and the following root definition was defined: “*Define a visualization that represents the narrative of the individual data of pregnancies present in the PMCP database and produce quality information that allows comparison and with a short learning curve*”.

The purpose of this iteration is to be able to show, through visualization, an individual narrative of pregnancies. This visualization should serve to understand or signal cases of reinfection, recurrence, or not performing enough exams. From this definition, several visual forms of data representation were analyzed and the Trigram was proposed.

### 3rd cycle - syphilis Trigram refactoring and infographic interface proposition

The third iterative development cycle focuses on the visualization, as isolated Trigrams were not a tool for analysis. It would be necessary a graphical interface that grouped the Trigrams and had filter tools. In addition, the usability issue in the accessibility aspect should be included in the visualization. As a result, in this third iterative development cycle, it was agreed the following root definition: “*Improve Trigrams, making them more accessible and with less eyestrain, and develop a Graphical User Interface (GUI) that supports them and allows refinement of information and filters, with easy comparison and a short learning curve.*”.

The main contributions of this cycle were:

- Creation of the Trigrams results matrix to represent the number of cases according to the Venereal Disease Research Laboratory (VDRL) results at birth and the mother’s last test;
- Development of the first version of the GUI containing the Trigrams and the Trigrams results matrix; and
- Trigrams improvement with color-adjusted, better for people with color vision problems, as well as with mild intensity to prevent eyestrain.

However, in the evaluation, it was noticed that the data provided did not have enough scale to explore and challenge the model proposed by the Trigrams. The number of occurrences was too low (n=21) to challenge the view scale. After the approval of this first prototype, it was agreed to expand the database with a larger segment of the SIS-MC database.

### 4th cycle - scaling data and visual element addition

This cycle begins with the availability of a more complete SIS-MC database for study, intending to validate whether the visualization is scalable. Thus, the data analysis process was restarted, now with a range of data covering all cities served by the PMCP, but keeping the cut of the years 2016 and 2017. The amount of data available for analysis jumps from 1,552 pregnancies to 43,938 pregnancies, with 217,444 prenatal care, 39,309 births, and 38,559 children in the new database made available by the PMCP. With the increase of the data scale, it was necessary to reassess the quality of the data proposed for visualization. Thus, the following root definition was agreed upon: “*Improve Trigrams through greater use of data, develop and validate its graphical interface allowing system actors to produce quality information, easy to compare and with a short learning curve*”.

Thus, the main contributions of this cycle were:

- Pre-processing of the new database with a larger number of cases for analysis and visualization;
- Addition of a new element to the Trigram to represent the gestational age at delivery, as well as the division of the Trigram into trimesters; and
- Adaptation of the graphical user interface to accommodate more viewing data, resulting in a density reduction with interface elements and adding navigational elements.

### 5th cycle - average Trigram proposition and interface refactoring

After increasing the scale of the data, the visualization system began to be validated closer to the actual use of stakeholders. The suitability of Trigrams to increase the data scale raises the need for new data selection, grouping, and analysis tools. In this cycle, new ways to expand the infographic space of information production will be developed. In addition, the language applied to visualization was not coherent with the common domain of the PMCP, as assessed in the previous cycle. During the process of specifying the root definition of this cycle, the adjustment of the representation language for the domain was considered. The way to visualize the data impressed the stakeholders, but the noise of the terms caused many problems in the evaluation. Thus, the following root definition was defined with stakeholders: “*Propose a data visualization of a location that maintains the Trigrams narrative, but in a grouped way, and standardize the terms of all interfaces with terms close to the PMCP domain.*”.

The main contributions of this cycle were:

- Change of terms that define the lack of data to a language closer to the reality of the stakeholders;
- Proposition of the Mean Trigram to connect, compare and synthesize the mean distribution of the narratives of three variables relevant to the context: first test, last test, and test in childbirth; and
- Graphical interface update with new elements, such as year selector and Average Trigram.

During the assessment, the relationship between prenatal care and the impact on syphilis care was raised. One of the PMCP’s premises is to encourage an increase in the amount of prenatal care. Being able to see, in the infographic interface, the relationship between successful or unsuccessful narratives and the amount of prenatal care would be important information for the stakeholder analysis.

### 6th cycle - average Trigram refactoring

In this iterative cycle, the greatest demand is an adjustment of the Mean Trigram for greater accuracy of data representation. And another increase in visualization is the relationship between prenatal care and the narrative of syphilis exams. It is necessary to evaluate the profusion of elements in the interface, so as not to add noise. The Mean Trigrams present an improved analysis of the data, but it is necessary to validate their accuracy and reliability for representation. The root definition defined in this cycle was: “*Improve the Mean Trigrams and the infographic view as a whole, considering the representation of prenatal care within the infographic interface.*”.

The main contributions of this cycle were:

- Visualization of prenatal care in the series of Trigrams, allowing comparison between test results and care for pregnant women; and
- Improvement of Trigram to visualize the information about the latest syphilis exams.

## Results

### The evolution of the Trigram

**Fig. 6:**
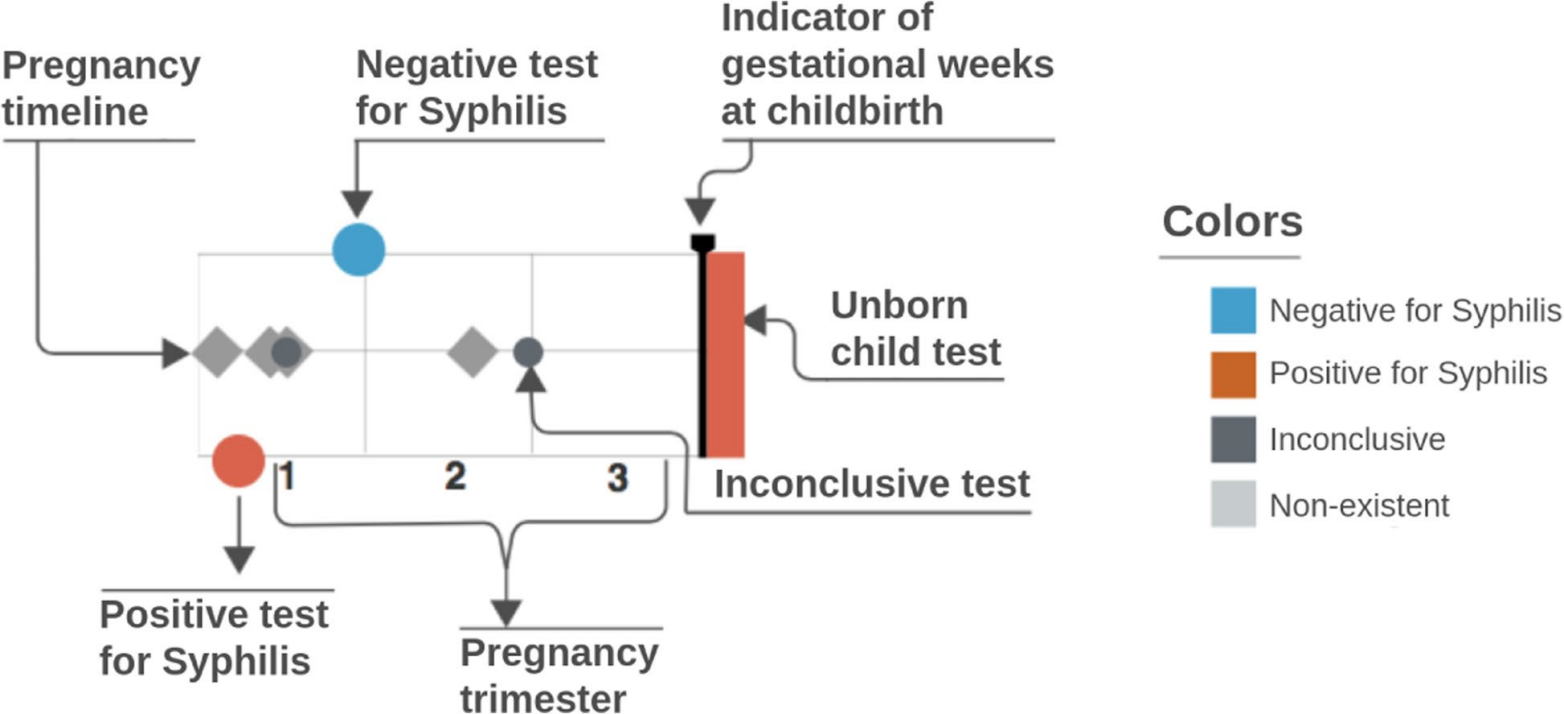
Visual structure of the Trigram

Based on the principles presented previously, an adequate representation for the problem domain called **Trigram** was proposed, shown in Fig. 6. Trigrams are an infographic representation of the narrative of a pregnancy through a time series. Each Trigram presents a comparable view of pregnancy in relation to syphilis considering tests and outcomes. By definition, it is a multivariate and interactive series that visually simply and comparable data from a pregnancy.

The Trigram was inspired in the form of a musical pentagram, and each element represented in the time series is positioned horizontally in time in relation to the birth date and the gestational weeks. On these lines, circles are positioned vertically related to the result of the pregnant woman’s syphilis tests, and can be classified as positive (circle positioned in the first line, from the bottom to the top), unavailable/null (second line), or negative (third line). The second line of the Trigram is also used as a basis for prenatal care, using diamonds. This representation proved to be resilient: they can be occluded by the examination, but they do not lose time series information. They can be grouped and, even with a few days of difference, they still show the quantity and its value in the time series.

At the end of the time series, there is a rectangle that represents the result of the outcome, indicated by a color, to reinforce the meaning and facilitate the user’s reading. The line with a small arrow pointing downwards represents the gestational age at delivery, to assess the loss or not of the gestational age. Within pregnancy care, the loss of gestational days or weeks is valuable information to guide the action of healthcare. A loss can severely impact the unborn child’s development [25].

The Trigram series is divided into three parts, each representing a trimester since the Ministry of Health of Brazil [26] recommends that the first syphilis test be performed in the first trimester of pregnancy and another one be repeated at the beginning of the third trimester if the results remain negative. In particular, the third trimester test is focused on reducing vertical transmission (mother-to-child transmission). Thus, users of the visual system can easily measure and compare the ratio of exams within the significant time series for the monitoring of pregnant women.

**Fig. 7:**
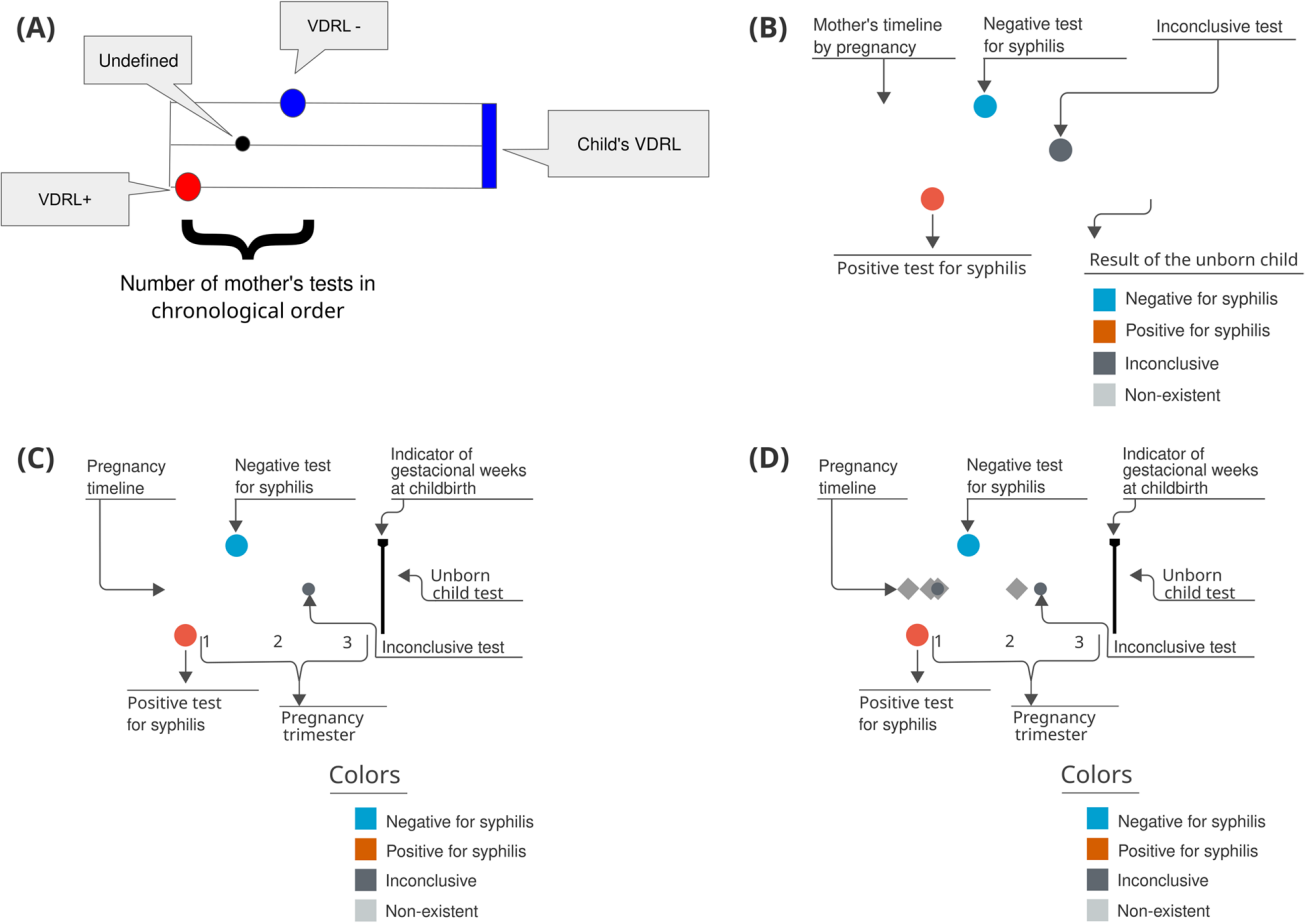
Evolution of the Trigram versions. Source: the author

However, the final design of the Trigram shown in Fig. 6 was based on cycles, as described in the previous section. Figure 7 shows the evolution of the Trigram.

You can see that several improvements were made during the development of Trigram. For example, in the 3rd cycle (B), the color pattern was adjusted. The chosen colors are safe for people with color vision problems and have a soft intensity to avoid eyestrain. Positive tests for syphilis are colored orange-red, negatives light blue, and undefined ones dark gray. When endpoints are classified as non-existent, it is colored with light gray. And to make it easier to read and reduce the learning curve, a Trigram color, and reading legend has been added.

Thus, a visual solution was designed that was easy to learn, was part of a common user repertoire (music), and supported the multivariable and temporal complexity of the data, allowing for quick comparison and interpretation of a pregnancy. One of the Trigram’s visual advantages is the composition of the pregnancy narrative. Previously, through the existing systems in the PMCP, the reports showed whether or not there was syphilis in a given pregnancy, but did not indicate when, nor how many times per pregnancy or if there was reinfection; important information for health surveillance. With Trigrams, it became possible to evaluate this narrative construction of pregnancy in relation to syphilis, including mapping optimal, ideal, and undesirable situations.

### The evolution of the mean Trigram

The grouped view comes to facilitate the pre-choice of data observation. It is difficult to get a general idea of a city from the individual Trigrams presented previously. The big challenge is how to represent it in a clear, comparable way and with the refinement needed by users. In the proposed Trigrams, the temporal representation of the exams helps to understand the relationship of the pregnancy narrative, considering reinfections, false negatives, and even care failures. For an aggregated view, it would be important to maintain this structure that presents the narrative of outcomes.

After studying the technical manuals for notification and surveillance of syphilis, [26] and [27], it was defined that the grouped view should understand the last phase of the narrative from pregnancy to the outcome. As one of the main actions that the visualization system proposes is to reduce the number of cases of congenital syphilis, analyzing the first and last exams during pregnancy would be a way to see how the treatment happened during prenatal care. If the first test is positive for syphilis, it is expected that there will be another test with a negative result. Also, if the last test is positive, there is a high chance of having congenital syphilis. Thus, concentrating on these two variables would enable a grouped synthesis of pregnancy. And, to close the whole narrative, the outcome must be represented. One of the expressive results of the assistance policy for pregnant women is the result of the test during childbirth. A positive test can indicate a lack of assistance, relapse, drug resistance, or even false negative. Thus, the visual representation must contain at least these three variables (first test, last test, and test at delivery) to synthesize the narrative of the Trigrams with the coarsest granularity.

**Fig. 8:**
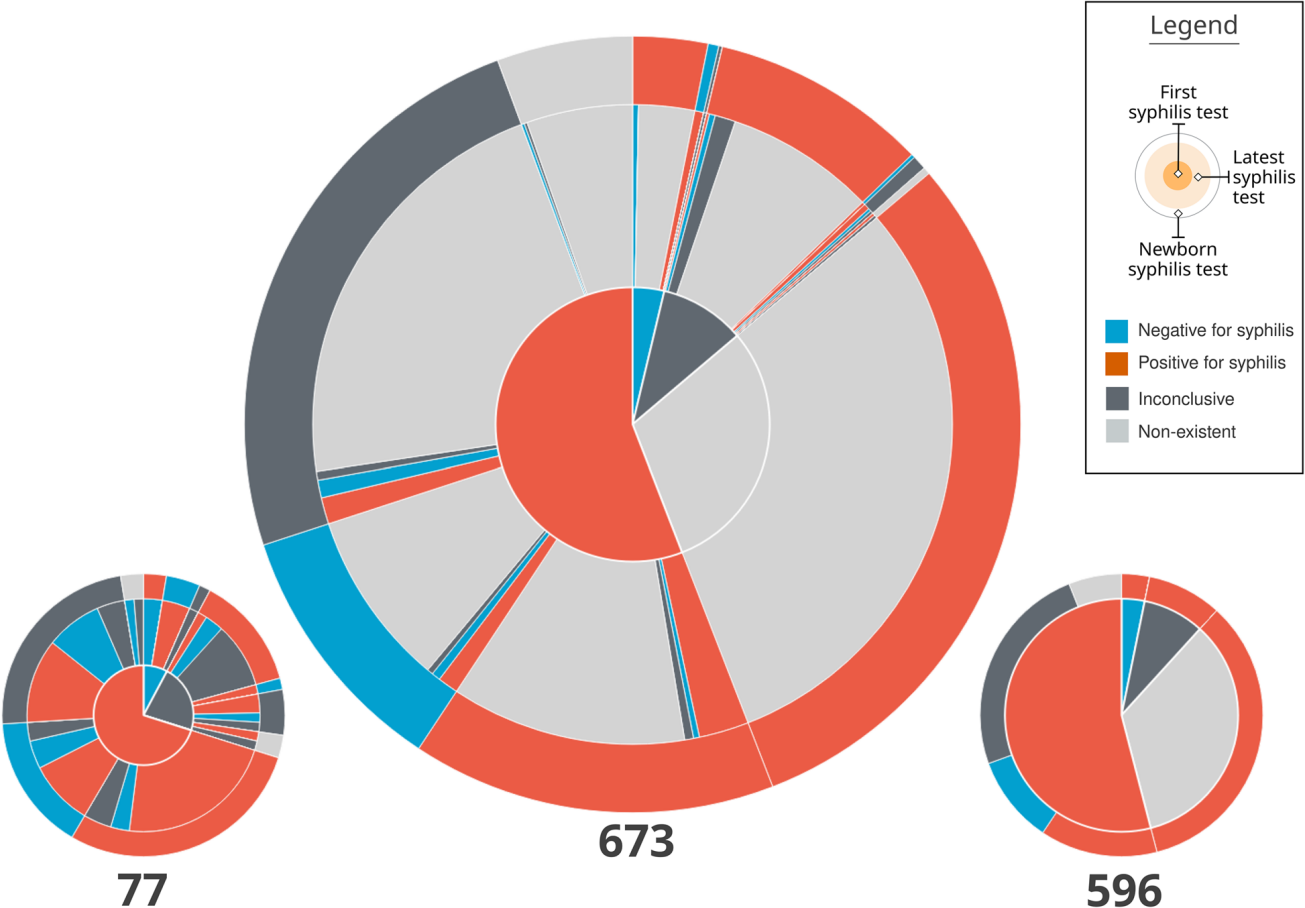
Mean Trigram expanded with two Mean sub-Trigrams. The Mean Trigram on the left represents pregnancies that have more than one test, and the right one only represents pregnancies with one test. Source: the author

The visual composition of a pie chart with three concentric rings was called **Mean Trigram** (Fig. 8). The main idea of this visual element is to synthesize the average distribution of the narratives among the three variables analyzed. Each sector is colored with the same Trigram color palette and keeping the same meaning as the exams. The innermost circle represents the pregnancies, the intermediate represents the most recent exams, and the outermost one the outcome.

The central Mean Trigram is the Mean Trigram of the location and year chosen by the user. The lower Mean sub-Trigram on the left represents a segment of pregnancies that have more than one test. And the lower Mean sub-Trigram on the right represents those who only have one test (that is why there is no intermediate ring between the pregnancy test and the outcome). Note that the number of pregnancies for each chart now represents a different amount. The ideal scenario is achieved when the majority of the cases are in the Mean sub-Trigram on the left, which is not the case for the analysis of the presented sample.

The central part of the chart is sectioned between mothers with at least one test confirming syphilis (represented in red) and mothers who do not have tests that confirm it (represented as blue, in the absence of a positive test for syphilis), but in both cases the mothers have syphilis. Then, these two initial sectors are directly related to their outcome, represented by the outermost ring. Thus, pregnancies with positive tests for syphilis are subdivided into an outcome with a positive test for syphilis, an outcome with a negative test for syphilis (ideal), an inconclusive outcome (with no data in the system), and no information (there is no record of the outcome in the database).

The sector of mothers who do not have a positive test for syphilis (blue sector in the middle of the Mean Trigram) is defined by its outcome, so all outcomes have a positive test for syphilis. Therefore, this group is also syphilitic: defined by the outcome at the time of delivery, due to the absence of positive tests before pregnancy.

Finally, the intermediate sector between pregnancies and outcomes, for each outcome section there is a representation for the last exam. Thus, it can be observed whether a pregnancy, that started as positive for syphilis, had the result changed before the outcome or if this situation was confirmed. In the case of mothers who did not have a positive test, the greatest information comes from false negatives (represented by blue), inconclusive data (dark gray), and lack of prenatal tests (represented by light gray).

**Fig. 9:**
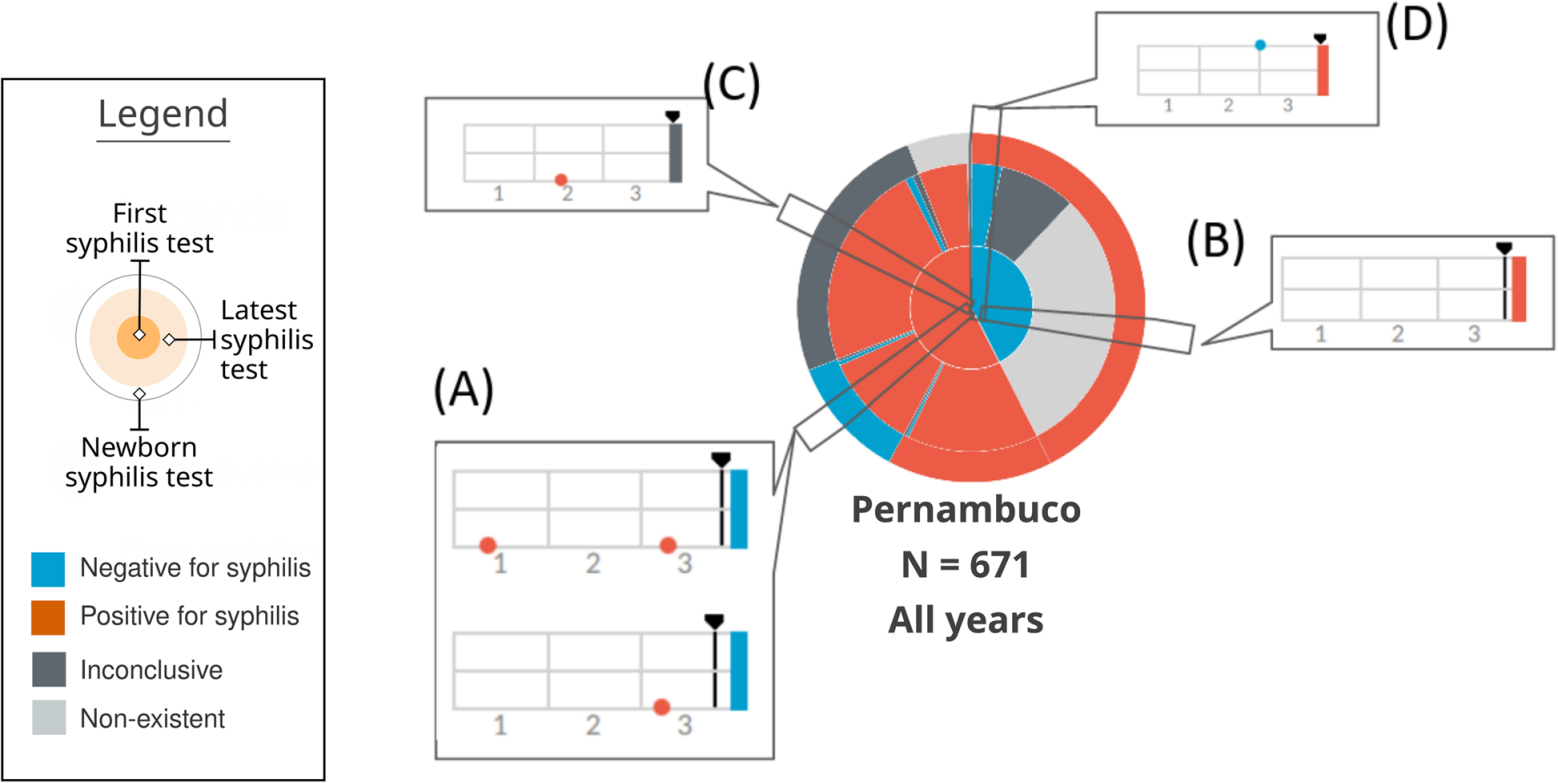
Reading of the Mean Trigram. Each transverse slice represents one or more Trigrams according to the three analyzed variables. Source: the author

Section (A) of Fig. 9 represents a case in which the mother had the first positive test, whether or not she had a second positive test, but the test at delivery was negative. Thus, it shows the number of mothers who were assisted by the health system but did not have a test to confirm the treatment. Section (B) of Fig. 9 represents a Trigram that has no test. This category was a domain discovery learned during the generation of the Mean Trigram. There is a large group of mothers who do not have any tests registered during pregnancy, but the test at birth indicates that she had syphilis. Section (C) of Fig. 9 shows a case in which there is an inconclusive outcome record for a pregnancy that had one or more positive tests for syphilis. And section (D) of Fig. 9 represents a false negative, serologic scarring, or infection very close to delivery. Thus, despite having a test that is proven to be negative for syphilis, the outcome has an unfavorable result, with a positive test for syphilis.

**Fig. 10:**
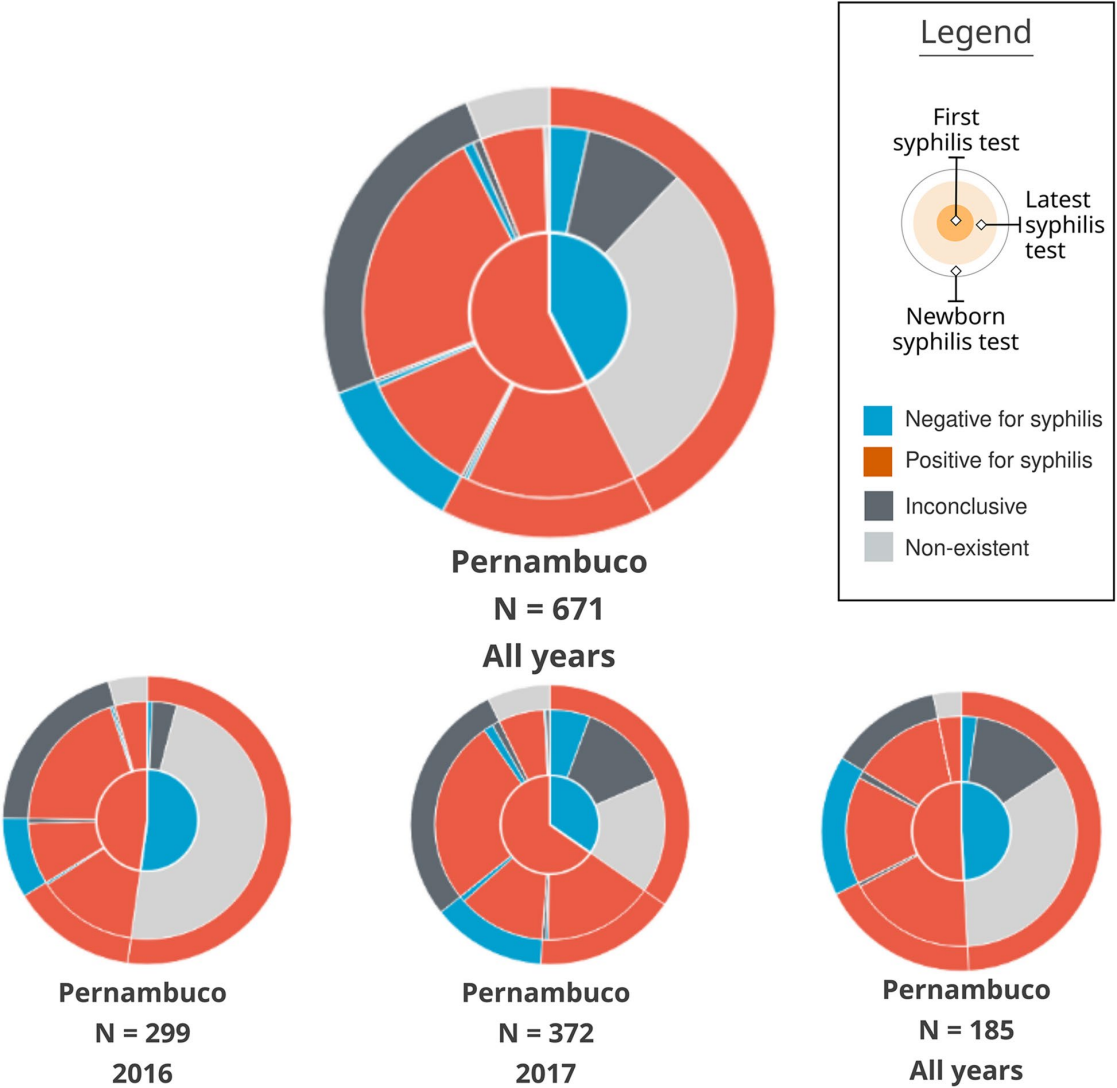
Comparison between Mean Trigrams of the State of Pernambuco, for different years and the city of Recife

One of the characteristics of the Mean Trigrams, in addition to the narrative and quantitative representation, is the power of comparison. The Mean Trigram allows comparison between regions and rapid assessment of the results of public policies over a given period of time. Thus, it serves as a tool for individual and comparative evolution (benchmarking). In Fig. 10, there is a comparison between the Mean Trigrams of the State of Pernambuco, by years and in total years with the city of Recife.

Note that, despite the different sample quantities, it is possible to evaluate the distribution of cases, considering the chosen time interval. A noteworthy piece of information is the increase in the number of false negatives (a negative test before delivery positive for syphilis). It is exactly a differential feature between non-treponemal (rapid test) and treponemal tests. Non-treponemal tests, within the syphilis treatment protocol [27], should be used for initial screening in pregnant women but confirmed with other treponemal tests. As a result, this section of the Trigram points to false negatives of rapid tests and, at the same time, the possible lack of tests with a more accurate technique.

### The evolution of the user interface

**Fig. 11:**
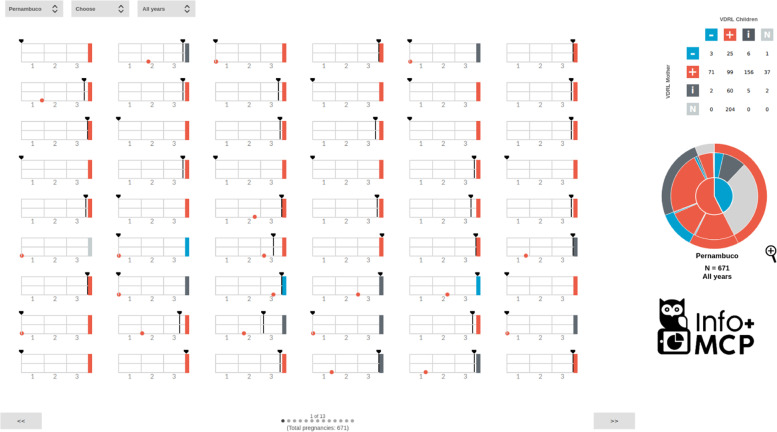
Complete infographic interface, with Mean Trigram. Source: the author

The complete infographic interface is shown in Fig. 11. There is a year selector to segment the data for visualization. The user can filter by city and year, and all Trigrams and Mean Trigrams interface will respond to these filters.

One of the important elements of visual clarity is the ability to compare Trigrams by vertical alignment. Thus, when choosing a column as an element of perception, it is possible to compare how the Trigrams are distributed in the test and gestational age with each other in the visualized sample. This comparative view, added to the selected filters of the last pregnancy test and the birth test, allows users to produce context information through a broad perception of the Trigrams. The comparative view is completed with a case-by-case focus on the narrative of each pregnancy by Trigram. Hence, there is a comparable infographic view of the situation in a given location.

There is also a **matrix of Trigram results**. Considering that one of the main goals of care for pregnant women with syphilis is to avoid the various damages caused by vertical transmission, it was decided to filter by the last pregnancy test and the birth test. The 4x4 matrix numerically represents the quantitative according to the following parameters: VDRL result at birth (positive, negative, indefinite, and non-existent) and the mother’s last test (positive, negative, and indefinite). In addition to the individual data of pregnancies in the Trigram, it is possible to consult the numerical quantity by comparing the last test and the outcome. This double view of the data reinforces the infographic approach, as the Trigrams show a finer granularity of pregnancies, while the matrix presents a development grouped by a combination of factors. This double vision improves the perception of reality in general and in the specific narrative. In this way, the analyst can filter whether or not the test result of the mother was transferred to the child at birth. In particular, this filter helps to understand cases of false negatives and failure to perform or report a test to prove the cure for syphilis. The test state indicators (represented by a “+”, “-”, “i”, and “n”) are interactive buttons that, when activated, filter the Trigrams by one variable at a time or a combination of two from different axes.

Furthermore, given the variation in the data, the Mean Trigram has, in the visualization interface, a zoom tool. Some slices in cities with more population or the total state have a representation that is difficult to perceive within the applied size of the Mean Trigram in the interface. With the zoom tool, the user can better understand these thin slices and use the mouse interaction over/hover for more information. In this version, the pagination system and matrix are already integrated with the complete SIS-MC base. And as said before, the matrix now also represents the Trigrams that don’t have any test, to be filtered in the execution.

## Discussions

One of the important factors of domain-specific infographics is their interactive and exploratory nature. Although normally presented separately, data visualization, interaction, and analysis are not mutually exclusive. Its integration is one of the main goals of data visualization [17]. Unlike the exploratory approach of [28], exactly because infographics are domain-specific that users can make good exploratory use of them. The exploitation is controlled within what is expected to be found. It’s not trial and error, it’s an investigation of situations that are within the scope of the domain, the data, and the views.

One of the potentials of domain-specific infographics is the ability to allow explanations of phenomena at a managerial and strategic level. It is not representing the data nominally, it is expanding the understanding of the data by comparing and relating the various visual elements. Far beyond the “what happened” is promoting a “how it happened” or “how to investigate what happened”. In this aspect, the infographic interface makes it possible to go from general epidemiological data to specific data on pregnancies.

As an example, the epidemiological bulletins of syphilis in Brazil in the years 2017 and 2018 were selected for analysis. These bulletins correspond to the data range of the PMCP database, referring to the years 2016 and 2017, respectively. Limitations of this analysis come from the form of notification of the Brazilian notifiable diseases information system, the SINAN, compared to the SIS-MC, since the SIS-MC data are not from all pregnant women in the State. On the other hand, the care provided by the PMCP is considered to be superior to the average SUS, due to the focus and reinforcement of care for pregnant women. So, the pregnant women analyzed in this comparison would be the best possible scenario in the SUS of the State.

The number of notifications of syphilis cases in pregnant women in the state in 2016 and 2017 were, respectively, 888 and 1,648. The PMCP assisted 141 and 235 pregnant women, respectively, in the same period. One of the problems of the comparison is that in SIS-MC the notification data are consolidated by pregnancy, but in SINAN, which serves as the basis for the bulletin, it is based on notifications. If the same pregnant woman has two positive tests during the same pregnancy, for SINAN it will count as two notifications, but in the SIS-MC it will appear as a pregnant woman with syphilis. This point will be important to understand how data is amplified by infographic analysis with Trigrams. Considering the unborn children with detection of syphilis less than 7 days (which is the PMCP notification interval), the number reported in the years 2016 and 2017 were respectively 1,465 and 1,860 notifications. Of this total, 13.5% and 10.2% were born at the PMCP. In this case, it can be attributed that the children are a part of the PMCP because there is no way to have more than one notification within 7 days.

**Fig. 12:**
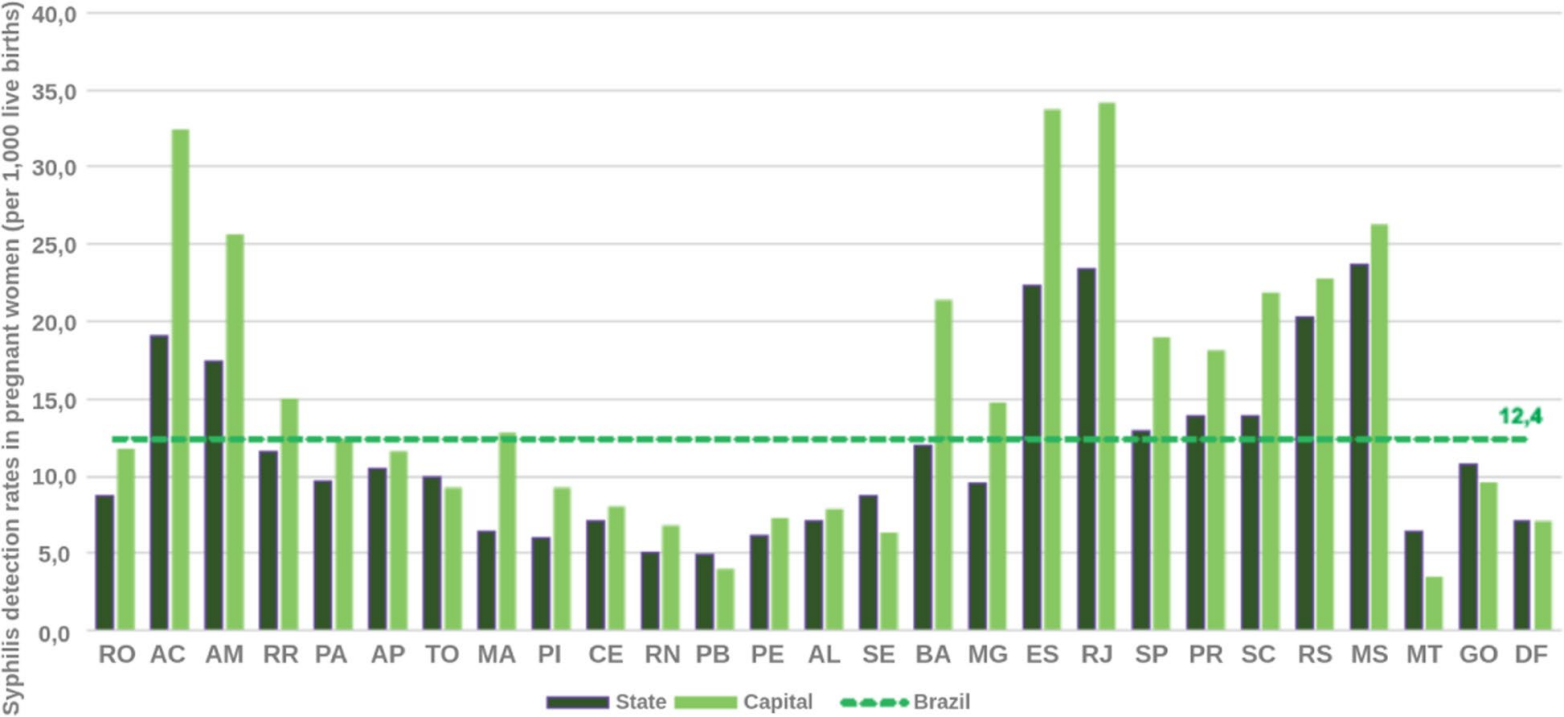
Syphilis detection rates in pregnant women according to Federation Unit and capital. Brazil, 2016. Source: SINAN, updated on 06/30/2017

**Fig. 13:**
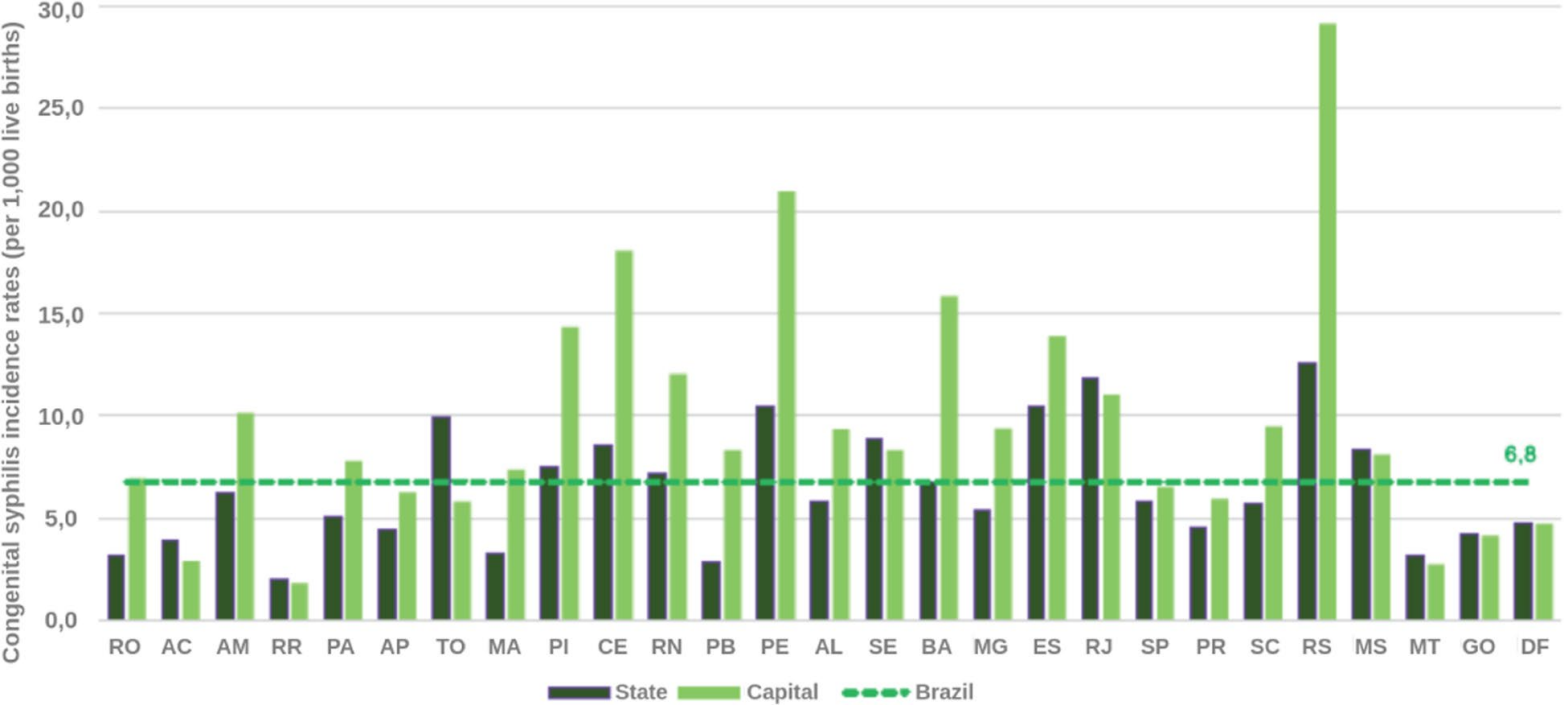
Congenital syphilis incidence rates by State and capital. Brazil, 2016. Source: SINAN, updated on 06/30/2017

**Fig. 14:**
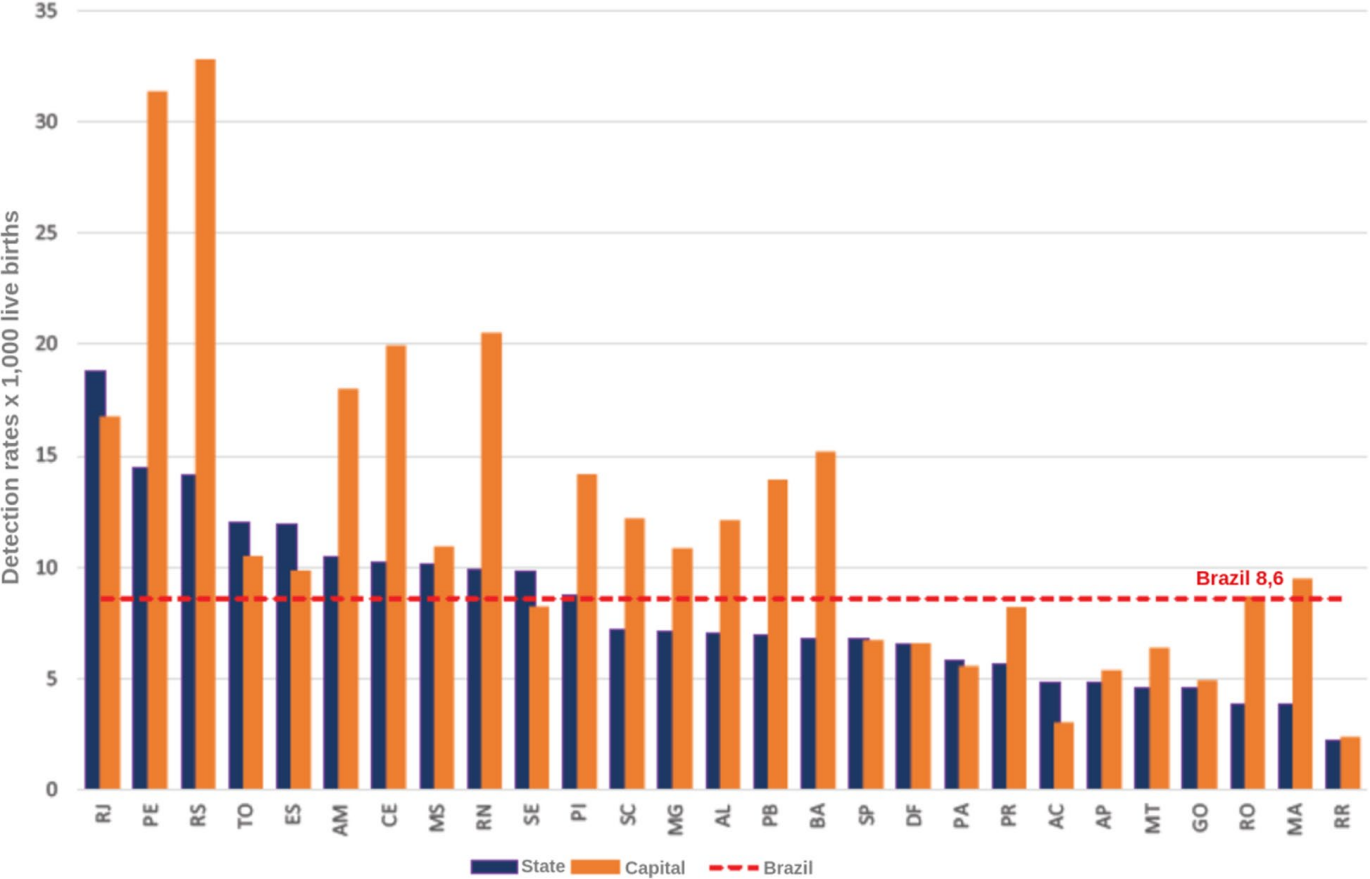
Congenital syphilis incidence rates by State and capital. Brazil, 2017. Source: SINAN, updated on 06/30/2018

**Fig. 15:**
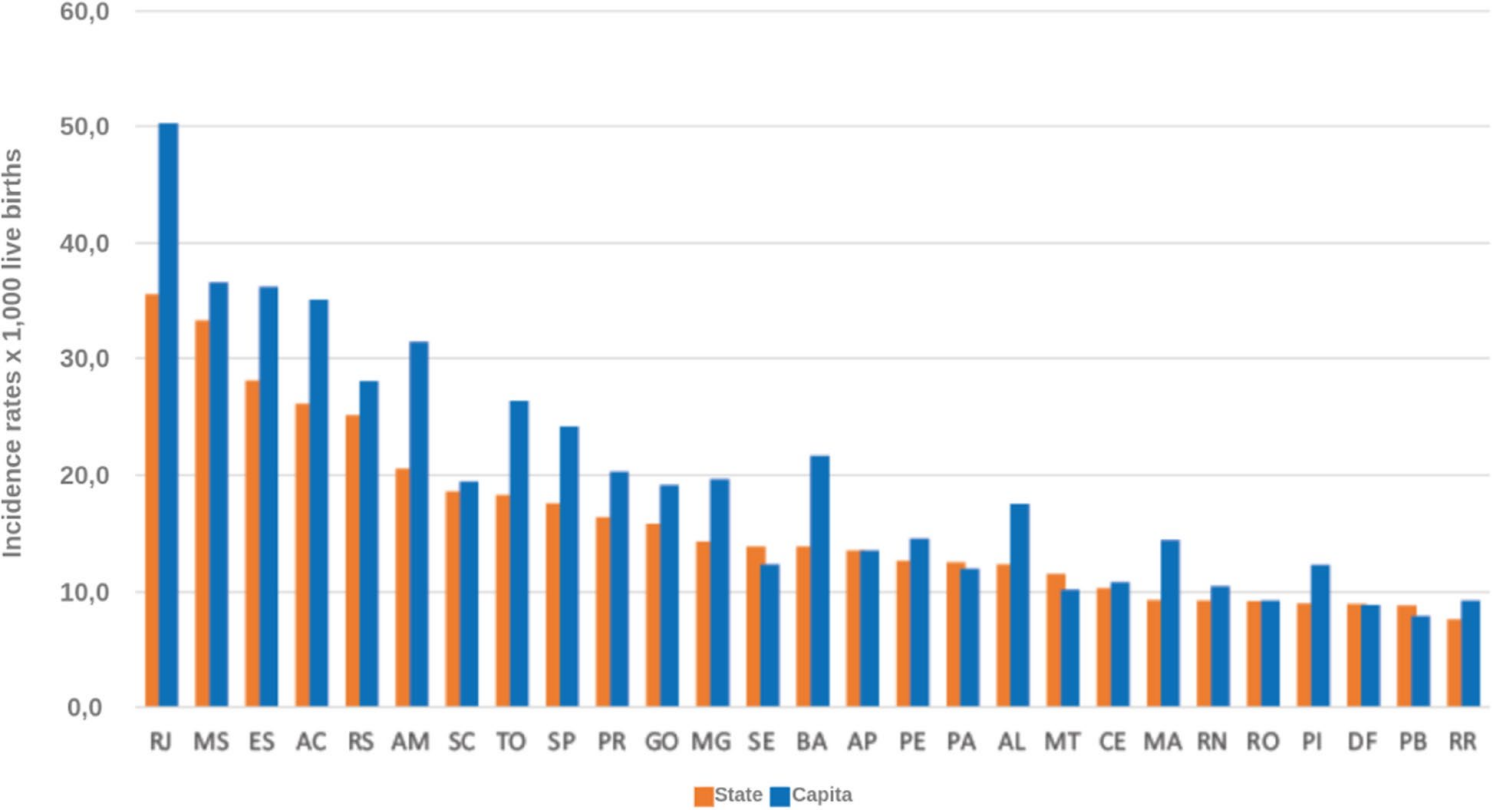
Syphilis detection rates in pregnant women according to State and capital. Brazil, 2017. Source: SINAN, updated on 06/30/2018

Two important pieces of information from the analyzed bulletins are the detection rate of gestational syphilis and the incidence rate of congenital syphilis. These rates represent the total number of notifications per thousand people in the population at the time. These are interesting rates because they are comparable over the years, as they are proportional to the population at the time. This information is presented in Figs. 12, 13, 14, and 15.

The syphilis’ protocol for pregnancy in Brazil recommends to perform testing at least two times during pregnancy, one at the first trimester and another at the beginning of the third trimester. It is important to state that every child and mother must have a test at birth. However, as the healthcare quality is marked by heterogeneity in Brazil, some pregnant women can have the first syphilis test only after labor. These two data are not directly superimposed because healthcare inequality in the context of the complexities of SUS. Taking this phenomena in consideration is one of the contributions of the Mean Trigram: the infographic presents the offset of these variables in the used dataset. Even if the numbers seem close or superimposed, the current situation in Brazil shows that there is a lack of testing and notification of gestational syphilis.

The detail is when comparing the rate between gestational syphilis and congenital syphilis. Boards below contain a comment on the relationship between these rates (highlighted in the board). At an epidemiological level, this relationship is indicative of a phenomenon. But, during the analysis of the Trigrams, it is noticed that it is a much more complex phenomenon.


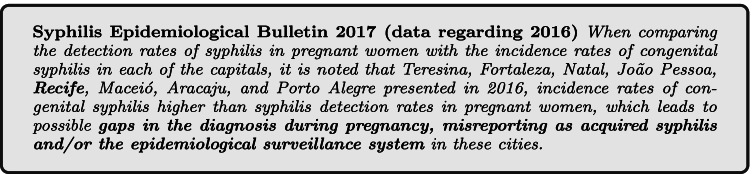

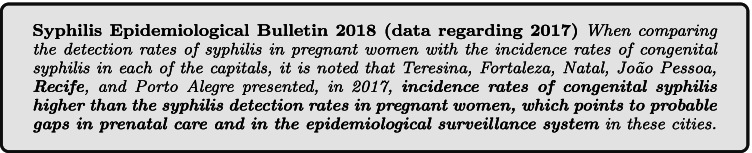


**Fig. 16:**
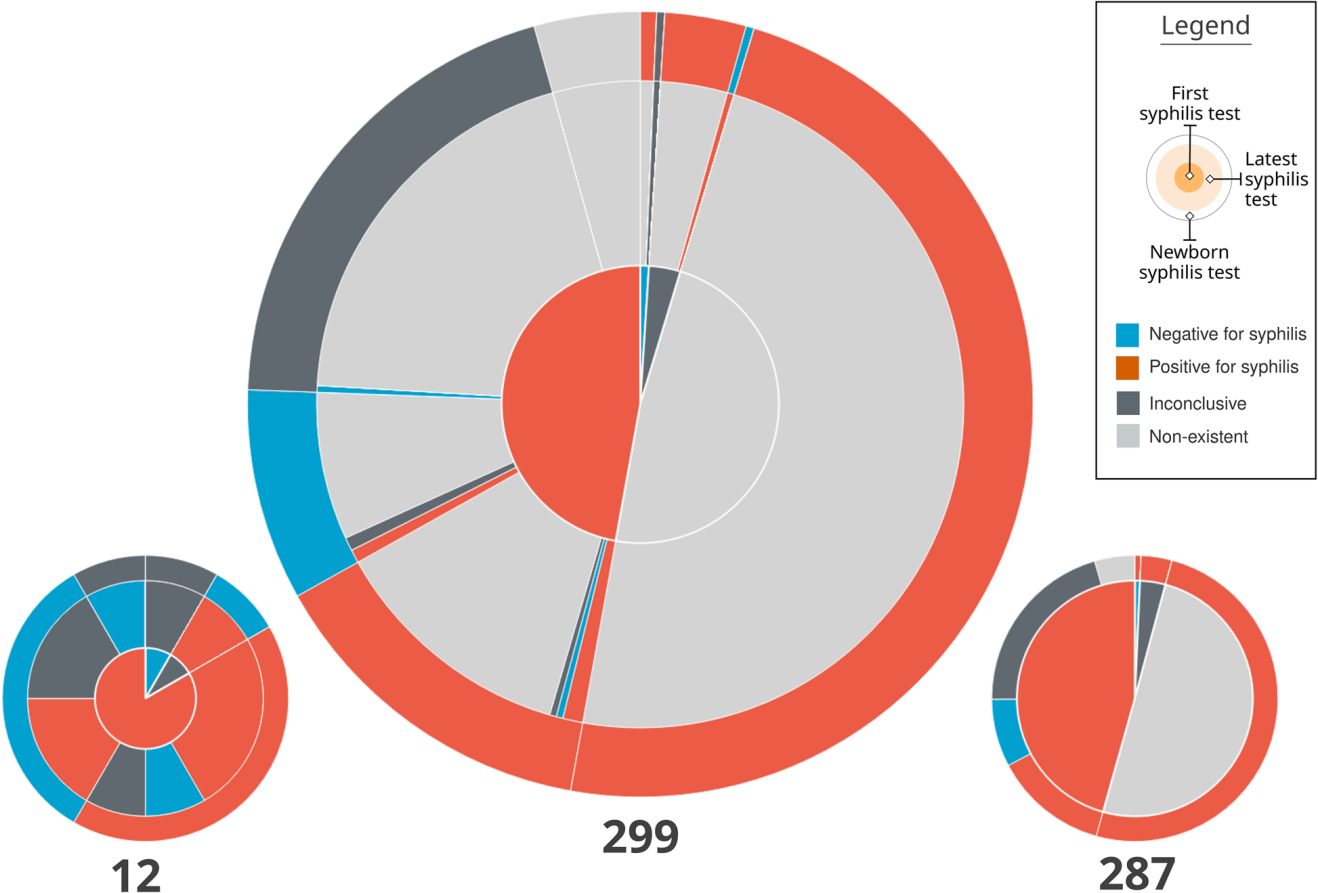
Mean Trigrams of the State of Pernambuco in 2016. Source: the author

**Fig. 17:**
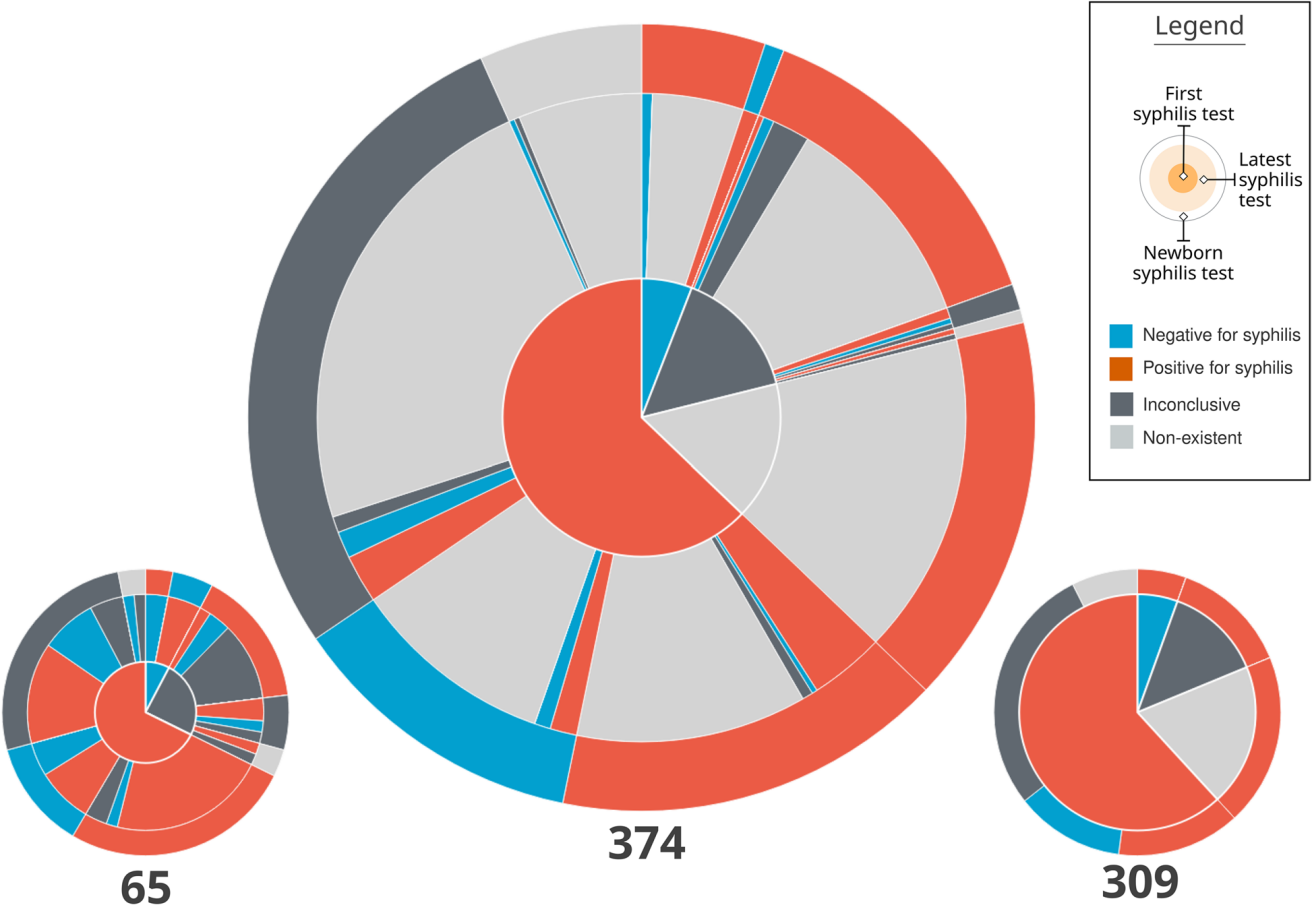
Mean Trigrams of the State of Pernambuco in 2017. Source: the author

Considering the limit of the analysis presented at the beginning of this section, the Mean Trigrams show a different viewpoint on the relationship between detection of gestational syphilis and the incidence of congenital syphilis. Figures 16 and 17 show the Mean Trigrams of the State of Pernambuco in the PMCP in 2016 and 2017. In the epidemiological bulletins, the syphilis detection rate in Pernambuco has more than doubled between 2016 and 2017 (from 6.1% to 12.6%). And this was reflected in the Trigrams, as the gray slice of the innermost circle significantly reduced. But the relationship between pregnant women diagnosed with syphilis and outcomes with syphilis did not change as significantly. The unfinished tests of the unborn child have a greater relationship with the detection rate than the rate of congenital syphilis. Both charts are at the management level, but the granularity of the Mean Trigrams allows us to understand, in addition to the phenomenon represented in the rates, others that happen around them.

For example, the relationship between rapid tests false negatives results and cases of gestational syphilis. In the Mean Trigram for the year 2017, there is a significant proportion of pregnant women who tested negative for syphilis but the outcome was unfavorable. This slice hardly appears in the 2016 Mean Trigram. The absence of confirmation tests, as provided for in the Technical Manual for the Diagnosis of Syphilis [27], is one of the most present factors in the notification of congenital syphilis. As explained previously, there is no direct relationship between pregnant women with syphilis and congenital syphilis. There is a misalignment between these two data in the samples analyzed in this work. Probably with more data and with the breadth of the entire state, this relationship should change. But within this universe of two years, the comparison is very clear.

The infographic analysis promoted by the infographic interface allows this alternation between broad epidemiological data for a case-by-case refinement. This vision and other implementations of this development also aim to meet the recommendations of the World Health Organization (WHO).

## Conclusions and future works

The Syphilis Trigram is an infographic that represents the complexity of gestational syphilis and congenital syphilis in a time series. The Syphilis Trigram allows analyzing the pregnancy narrative for cases of gestational syphilis, representing in a comparable way the relationship between prenatal care, exams and pregnancy outcome with or without congenital syphilis. Aided by other visual elements, Mean Trigram and selection matrix, it composes an infographic interface for the analysis of gestational syphilis and congenital syphilis at the managerial and information executive level.

The main contribution of this work is the proposition of infographics in a specific domain for health. These infographics are quantitative representations that allow, through a visual synthesis, to understand a health phenomenon in both macro and micro scales. The great advantage of this perspective is to allow this change of scope (macro and micro) in the same visual context to help users investigate complex phenomena through consistent and comparable views. So, in addition to having a general idea about a sample, users can see and understand the individual motivators that might influence the overall result. The visualization enabled production of new information and forms of analysis for stakeholders, allowing for a greater and better understanding of their data.

This work proposed a contribution to the Brazilian SUS analysis and decision-making process through data visualization. The contributions presented are far from being the definitive solution for the SUS process, but it is believed that there is an increase in the way of producing information in some specific spheres, with even greater possibilities for expansion. But, in the specific domain in which they were designed, these visualizations can definitely help to a better health care system, optimize resources, and be used as a learning tool to avoid past mistakes.

The continuation of similar researches can, even in small steps, greatly improve the quality of the SUS. And considering the complexity of the SUS and the reality of Brazil, any improvement has the potential to positively impact the lives of Brazilians. We also plan to make a new analysis considering the latest epidemiological bulletin, once the patterns of incidence rates of congenital and gestational syphilis changed in most of the states of Brazil. By using Trigram, we could show that there is not a superimposition.

## Data Availability

The data that support the findings of this study are available from *Programa Mãe Coruja Pernambucana* (PMCP) but restrictions apply to the availability of these data, which were used under license for the current study, and so are not publicly available. Data are however available from the authors upon reasonable request and with permission of Judith Kelner (e-mail: jk@cin.ufpe.br).

## Abbreviations

UN: United Nations
SINAN: *Sistema de Informação de Agravo de Notificação*
STI: Sexually Transmitted Infections
PHC: Primary Health Care
PMCP: *Mãe Coruja Pernambucana* Program
OAS: Organization of American States
SUS: *Sistema Único de Saúde*
HIV: Human Immunodeficiency Virus
SIS-MC: *Sistema de Informação do Mãe Coruja*
GUI: Graphical User Interface
VDRL: Venereal Disease Research Laboratory
WHO: World Health Organization
SIM: *Sistema de Informação sobre Mortalidade*
CATWOE: Customers, Actors, Transformation process, Weltanschauung, Ownership, and Environmental constrains

## Footnotes

1 https://maecoruja.pe.gov.br/cantos-mae-coruja/
